# Multilevel analysis of determinants in postnatal care utilisation among mother-newborn pairs in India, 2019–21

**DOI:** 10.7189/jogh.14.04085

**Published:** 2024-05-10

**Authors:** Sohee Jung, Hyejun Chi, Yun-Jung Eom, S.V. Subramanian, Rockli Kim

**Affiliations:** 1 Interdisciplinary Program in Precision Public Health, Department of Public Health Sciences, Graduate School of Korea University, Seoul, Republic of Korea; 2 Harvard Center for Population and Development Studies, Cambridge, Massachusetts, USA; 3 Department of Social and Behavioral Sciences, Harvard T.H. Chan School of Public Health, Boston, Massachusetts, USA; 4 Division of Health Policy and Management, College of Health Sciences, Korea University, Seoul, Republic of Korea

## Abstract

**Background:**

Postnatal care (PNC) utilisation within 24 hours of delivery is a critical component of health care services for mothers and newborns. While substantial geographic variations in various health outcomes have been documented in India, there remains a lack of understanding regarding PNC utilisation and underlying factors accounting for these geographic variations. In this study, we aimed to partition and explain the variation in PNC utilisation across multiple geographic levels in India.

**Methods:**

Using India’s 5th National Family Health Survey (2019–21), we conducted four-level logistic regression analyses to partition the total geographic variation in PNC utilisation by state, district, and cluster levels, and to quantify how much of theses variations are explained by a set of 12 demographic, socioeconomic, and pregnancy-related factors. We also conducted analyses stratified by selected states/union territories.

**Results:**

Among 149 622 mother-newborn pairs, 82.29% of mothers and 84.92% of newborns were reported to have received PNC within 24 hours of delivery. In the null model, more than half (56.64%) of the total geographic variation in mother’s PNC utilisation was attributed to clusters, followed by 26.06% to states/union territories, and 17.30% to districts. Almost 30% of the between-state variation in mother’s PNC utilisation was explained by the demographic, socioeconomic, and pregnancy-related factors (i.e. state level variance reduced from 0.486 (95% confidence interval (CI) = 0.238, 0.735) to 0.320 (95% CI = 0.152, 0.488)). We observed consistent results for newborn’s PNC utilisation. State-specific analyses showed substantial geographic variation attributed to clusters across all selected states/union territories.

**Conclusions:**

Our findings highlight the consistently large cluster variation in PNC utilisation that remains unexplained by compositional effects. Future studies should explore contextual drivers of cluster variation in PNC utilisation to inform and design interventions aimed to improve maternal and child health.

The Sustainable Development Goal (SDG) 3 calls for a reduction of the global maternal mortality ratio to less than 70 per 100 000 live births [1] and neonatal mortality rate to at least as low as 12 per 1000 live births by 2030 [2]. Approximately 30% of maternal deaths occur due to severe bleeding after childbirth, infections, high blood pressure during pregnancy, and complications from delivery [3–6]. Newborns also face the highest risk of mortality in the first month of birth due to intrapartum-related complications, infections, and birth defects [7,8]. These excessive deaths are largely preventable with basic health services and medical interventions in the postnatal period [5,9,10], making postnatal care (PNC) an essential component of health services for maternal and newborn health [3].

The World Health Organization (WHO) recommends all mothers and newborns to receive PNC within 24 hours of birth, followed by at least three additional postnatal examinations within 48–72 hours, between 7–14 days, and six weeks after birth [3]. The latest guideline particularly emphasises the critical importance of the first day of birth in preventing haemorrhage and hypertension, which together account for about 40% of maternal deaths [3]. Ideally, all mothers should be assessed for vaginal bleeding, uterine contraction, fundal height, temperature and heart rate, blood pressure, and urine void during the first PNC [3]. As for newborns, the current guideline recommends to check for any jaundice in the first 24 hours, followed by checks for feeding practices, history of convulsions, fast breathing, severe chest in-drawing, lack of spontaneous movement, fever, and low body temperature during subsequent PNC visits [3]. Given the distinct list of postnatal examinations required for mothers and newborns, it is imperative to monitor the coverage for both populations jointly and independently.

In India, despite the rapid economic growth, maternal and neonatal mortality ratios remain high, with 97 maternal deaths per 100 000 live births in 2018–20 and 20 neonatal deaths per 1000 live births in 2020 [11]. Moreover, the benefits from economic growth have been unevenly distributed across India, resulting in substantial geographic and social inequality in diverse health outcomes [12–14]. Health policies and interventions planned, implemented, and monitored at state and district levels – while overlooking the local contexts – have also widened small area variation in various maternal and child health outcomes [15–21]. For example, prior multilevel studies found significant variations within districts in the burden of child undernutrition [16] and caesarean delivery [17], highlighting the need to identify high risk groups more precisely to allocate resources in an equitable and efficient manner.

According to a recent study evaluating the geographic variations in a composite score of maternal health care quality, including PNC utilisation, more than half (58.3%) of the total geographic variance in India was attributed to clusters [18], indicating the importance of smaller geographic levels for addressing inequalities in maternal and newborn health. However, this study did not assess the extent to which the observed geographic variation at multiple levels can be explained by various known risk factors. Variation in health outcomes is a manifestation of compositional and contextual effects that lead to distinct policy implications [22]. Here, compositional effects refer to the clustering of individuals with similar characteristics that, in aggregate, result in differences between areas, while contextual effects refer to the social and built environment of geographic area themselves [22,23]. For instance, variation in PNC utilisation can be explained by concentration of low-income households in those areas (i.e. compositional effect) or lack of high-quality health infrastructure (i.e. contextual effect), or likely an interaction between them. A strong compositional effect indicates the need to intervene at the individual level, while a strong contextual effect indicates the need to intervene and modify more structural conditions. Despite their critical implications for policy discourse, the extent to which variation in PNC utilisation is driven by contextual vs compositional effects remains largely underexplored.

To date, most epidemiological studies have examined the risk factors of PNC utilisation at individual level only [24,25], without simultaneously considering their impact on other geographic units with administrative and political significance in India. Moreover, there remains a need to assess how the patterns in geographic variability in PNC utilisation vary across states/union territories given the known demographic, social, and economic heterogeneity. Considering this literature gap, we wanted to examine the following questions based on the latest nationally representative data in India:

1. Which geographic level (states/union territories, districts, or clusters) is relatively more important in PNC utilisation among mother-newborn pairs in India?

2. To what extent can various demographic, socioeconomic, and pregnancy-related predictors explain the contextual variation in PNC utilisation at each geographic level?

3. Is there a meaningful heterogeneity in geographic partitioning and explainability in PNC utilisation across states/union territories?

## METHODS

### Data source

We used data from the 5th National Family Health Survey (NFHS-5), which is equivalent to the Indian Demographic and Health Survey (DHS), for cross-sectional multilevel analysis [26]. The design of the NFHS-5 followed a two-stage cluster sampling frame, enabling estimations across 707 districts and 36 states/union territories in India. In the first stage of selection, primary sampling units composed of villages in rural areas and census enumeration blocks in urban areas were selected within each district. At the second stage, a fixed number of 22 households were selected from each primary sampling unit using an equal probability systematic sampling. A more detailed description is available elsewhere [26].

### Study population

We constructed mother-newborn pairs by merging the individual women’s file and children’s file from NFHS-5 (Figure S1 in the **Online Supplementary Document**). A total of 724 115 women of childbearing age (15–49 years) were surveyed, of whom 176 877 who had births in the last five years were considered eligible. After merging the data of women and children, we excluded children who were not alive at the time of survey (1.90%), not of the latest birth (24.08%), and not delivered at health facilities (9.28%). After we excluded observations with missing data on the number of antenatal visits (1.31%), we arrived at a final analytic sample of 149 622 mother-newborn pairs.

### Geographic levels

This analysis included four levels of geography, each with political, administrative, social, and cultural significance that can influence population health and well-being in India. States/union territories (level 4) are the highest political units at which the Indian government operates federal policies. Many states in India are more populous than a typical country and have distinct traditions, social norms, values, and trajectories of economic development [27]. Different levels of urbanisation, population density, and the proportion of scheduled castes/scheduled tribes across states/union territories have impacted their overall development [28]. For instance, states/union territories with a higher proportion of disadvantaged castes tend to have weaker social and financial infrastructure due to the historical economic deprivation and social exclusion [28]. Districts (level 3) are the lowest administrative level, with an average of 1.3 million rural population each [15]. The Indian government’s health policies and interventions are mostly conducted at the district level. For instance, Janani Suraksha Yojana, a conditional cash transfer programme that incentivises mothers to receive antenatal care (ANC) and give births in public health facilities, has been implemented at the district level [29]. Primary sampling units (level 2) are the smallest units in this analysis, representing the local environments in which mothers and newborns live. Primary sampling units are equivalent to communities/villages in rural areas and survey blocks in urban areas [26]; we will collectively refer to them as ‘clusters’ in this manuscript. Residents within the same cluster are likely to share similar level of socioeconomic status, gender norms, and accessibility to primary health centres, all of which can induce between-cluster inequality in maternal health care utilisation.

### Outcomes

The key outcome variable was PNC by skilled health personnel (i.e. doctor, auxiliary nurse midwife, nurse, midwife, lady health visitor, and other health personnels) within 24 hours of birth for mothers and newborns, respectively. For mothers, their experience of first PNC utilisation was identified using two questions: ‘Did anyone check on your health while you were still in the facility?’ and ‘How long after delivery did the first check take place?’ We constructed a binary indicator following the WHO recommendation, with ‘1’ coded for mothers who received their first PNC within 24 hours of delivery and ‘0’ otherwise. To identify newborns’ PNC utilisation, we used responses to the following questions: ‘Did anyone check on (NAME)’s health while you were still in the facility?’ and ‘How long after delivery was (NAME)’s health first checked?’ Based on their responses, we constructed a binary indicator of newborns’ PNC utilisation with ‘1’ coded for those who received their first PNC within 24 hours of birth, and ‘0’ otherwise.

### Predictors

We identified 12 important demographic, socioeconomic, and pregnancy-related correlates of maternal and child health service utilisation from prior literature [24,30–41]. For demographic characteristics, we included respondent’s current age (15–19 years, 20–34 years, 35–49 years), marital status (never married/widowed/divorced/separated, and married), current age of the child (<6 months, 6–23 months, ≥24 months), and sex of the child (male, female). In view of socioeconomic characteristics, we extracted factors relevant for maternal health care utilisation, such as maternal education (no education, primary, secondary, higher), household wealth quintiles constructed based on the household’s ownership of consumer items as well as dwelling characteristics (poorest, poorer, middle, richer, richest), health insurance (no, yes), type of residence (urban, rural), and religion (Hindu, Muslim, Christian, others). Lastly, pregnancy-related characteristics included birth order number (1st, 2nd, ≥3rd), number of ANC visits (<4 visits, 4–7 visits, ≥8 visits), and place of delivery (public facility, private facility). We used three categories of ANC visits to reflect the older (≥4 visits) and the newer (≥8 visits) WHO guidelines [3].

### Statistical analysis

#### Descriptive analysis

We first explored the distribution of the study sample and PNC utilisation within 24 hours of birth by each of the predictors for mothers and newborns separately. We included sampling weights in all descriptive analyses to account for the differential probabilities of participation and selection.

#### Four-level random intercept logistic models

Given the multistage sampling design, we estimated a series of four-level random intercept logistic models for the probability of mother/newborn *i* (level 1) in cluster *j* (level 2), district *k* (level 3), and state *l* (level 4) utilising PNC within 24 hours of birth as:

*logit*(*Pr*(*y_ijkl_* = 1*|X*)) = *β_0_* + (*u_0jkl_ + v_0kl_ + f_0l_*)

The random effects are interpreted as residual differentials for cluster *j* (*u_0jkl_*), district *k* (*v_0kl_*), and state *l* (*f_0l_*). These residuals are assumed to be independently and normally distributed with a mean of 0 and a variance of:



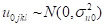





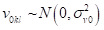





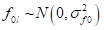



Hence, this model quantifies the following variances in the outcome:



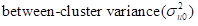





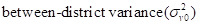





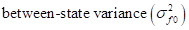



Individual-level variance cannot be calculated directly for a binary outcome; instead, a fixed variance from a logistic distribution is assumed as π^2^/3 = 3.29 [42]. For our first research question, we aimed to evaluate the relative importance of different geographic units when simultaneously considered. To do this, we calculated the proportion of the variation in the log odds of PNC utilisation attributable to each level, known as variance partitioning coefficient (VPC). For instance, the proportion of variation in PNC utilisation for cluster level was calculated as:



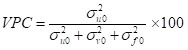



We excluded level 1 variance from the calculation of the total variation because of our focus on partitioning geographic variation, in line with prior studies [17–21,43].

### Model adjustment

For our second research question, we aimed to assess how much of the geographic variation in PNC utilisation can be explained by various compositional characteristics. For this, we made a series of step-wise adjustments to the four-level random intercept logistic models. A null model without any covariate adjustment provided the baseline crude variation at each level (model 1). We then added demographic variables (i.e. maternal age, marital status, and child’s age and sex) in model 2; socioeconomic variables (i.e. maternal education, household wealth, health insurance, type of residence, and religion) in model 3; and pregnancy-related correlates (i.e. birth order number, number of ANC visits, and place of delivery) in model 4. We assessed the relative importance of each covariate in terms of odds ratios (ORs), and 95% confidence intervals (CIs), as well as variance explained compared to the null model. Specifically, we calculated the proportional change in variance by comparing the geographic variance at each level before and after adjusting for the covariates.

### Stratified analyses

For our last research question, we performed stratified analyses to assess the consistency in main findings across 36 states/union territories. For state-specific analyses, we estimated the variation in PNC utilisation attributable to districts and clusters, as well as variance explained by the same set of predictors. We excluded states with fewer than six districts, including Andaman and Nicobar Islands, Chandigarh, Goa, Ladakh, Lakshadweep, Dadra and Nagar Haveli and Daman and Diu, Puducherry, and Sikkim, from state-specific analyses due to estimation issues. Lastly, we excluded Kerala and Himachal Pradesh, with little variation in PNC utilisation, for similar reasons, as well as Mizoram in the mother’s model and Andhra Pradesh in the newborn’s model.

We set the significance level for all statistical analyses at *P* < 0.05, using two-tailed tests. For descriptive analysis, we reported *P*-values for statistical significance of χ^2^ tests. We reported the results of the multilevel analysis with ORs and 95% CIs. We performed all analyses in Stata, version 16 (StataCorp LLC, College Station, TX, USA) [44] and MLwiN, version 3.05 (Centre for Multilevel Modelling, University of Bristol, UK) (using *runmlwin*) [45].

## RESULTS

### Distribution of PNC utilisation

In our analytic sample of 149 622 mother-newborn pairs, 82.29% of the mothers received PNC within 24 hours after delivery, a little lower than the prevalence of newborns (84.92%) (**Table 1**, **Table 2**). Compared to mothers who did not receive PNC within 24 hours of birth, mothers who received PNC were more likely to have higher than secondary education (19.99% vs 14.37%), have health insurance (25.70% vs 19.40%), have had eight or more ANC visits (23.11% vs 12.53%), and have delivered at private facilities (32.97% vs 23.46%) (**Table 1**). The proportion of newborns receiving PNC showed similar trends for most selected characteristics, except for birth order (**Table 2**).

**Table 1:** Distribution of mother’s PNC utilisation by demographic, socioeconomic, and pregnancy-related variables in India, NFHS 2019–21

	Total, n (%)	PNC utilisation, n (%)	No PNC utilisation	*P*-value*
**Demographic characteristics**				
Maternal age in years				<0.05
*15–19*	4106 (3.09)	3215 (2.84)	891 (4.25)	
*20–34*	130 745 (88.27)	108 348 (88.51)	22 397 (87.18)	
*35–49*	14 771 (8.63)	12 077 (8.65)	2694 (8.57)	
Marital status				≥0.05
*Never-married/widowed/divorced/separated*	2197 (1.16)	1788 (1.15)	409 (1.20)	
*Married*	147 425 (98.84)	121 852 (98.85)	25 573 (98.80)	
Current age of child in months				≥0.05
*<6*	18 516 (12.19)	15 402 (12.18)	3114 (12.22)	
*6–23*	54 769 (36.89)	45 246 (36.78)	9523 (37.41)	
*≥24*	76 337 (50.92)	62 992 (51.04)	13 345 (50.37)	
Sex of child				≥0.05
*Male*	80 366 (53.87)	66 501 (53.87)	13 865 (53.89)	
*Female*	69 256 (46.13)	57 139 (46.13)	12 117 (46.11)	
**Socioeconomic characteristics**				
Education				<0.05
*No education*	25 850 (16.43)	20 046 (15.18)	5804 (22.25)	
*Primary*	16 996 (11.14)	13 671 (10.78)	3325 (12.79)	
*Secondary*	81 615 (53.43)	68 122 (54.04)	13 493 (50.59)	
*Higher*	25 161 (19.00)	21 801 (19.99)	3360 (14.37)	
Household wealth				<0.05
*Poorest*	31 912 (19.50)	24 528 (17.49)	7384 (28.84)	
*Poorer*	33 557 (20.58)	26 807 (19.75)	6750 (24.44)	
*Middle*	30 846 (20.33)	25 750 (20.65)	5096 (18.83)	
*Richer*	28 721 (20.52)	24 647 (21.45)	4074 (16.20)	
*Richest*	24 586 (19.07)	21 908 (20.66)	2678 (11.69)	
Health insurance				<0.05
*No*	107 711 (75.41)	88 007 (74.30)	19 704 (80.60)	
*Yes*	41 911 (24.59)	35 633 (25.70)	6278 (19.40)	
Type of residence				<0.05
*Urban*	34 635 (29.76)	29 702 (30.99)	4933 (24.04)	
*Rural*	114 987 (70.24)	93 938 (69.01)	21 049 (75.96)	
Religion				<0.05
*Hindu*	113 329 (80.46)	94 861 (80.71)	18 468 (79.30)	
*Muslim*	20 941 (15.11)	16 864 (14.69)	4077 (17.10)	
*Christian*	8996 (1.97)	6883 (2.05)	2113 (1.60)	
*Others*	6356 (2.46)	5032 (2.56)	1324 (1.99)	
**Pregnancy-related characteristics**				
Birth order number				<0.05
*1st*	54 060 (36.39)	45 046 (36.82)	9014 (34.39)	
*2nd*	54 633 (37.42)	45 711 (37.89)	8922 (35.25)	
*≥3rd*	40 929 (26.19)	32 883 (25.30)	8046 (30.36)	
Antenatal visits				<0.05
*<4*	57 210 (37.60)	43 233 (34.11)	13 977 (53.83)	
*4–7*	62 631 (41.16)	53 831 (42.78)	8800 (33.64)	
*≥8*	29 781 (21.24)	26 576 (23.11)	3205 (12.53)	
Place of delivery				<0.05
*Public facility*	110 797 (68.71)	89 789 (67.03)	21 008 (76.54)	
*Private facility*	38 825 (31.29)	33 851 (32.97)	4974 (23.46)	
**Total**	149 622 (100)	123 640 (82.29)	25 982 (17.71)	

PNC – postnatal care

*Two-sample χ^2^ tests.

**Table 2:** Distribution of newborn’s PNC utilisation by demographic, socioeconomic, and pregnancy-related variables in India, NFHS 2019–21

	Total, n (%)	PNC utilisation, n (%)	No PNC utilisation, n (%)	*P*-value*
**Demographic characteristics**				
Maternal age in years				<0.05
*15–19*	4106 (3.09)	3398 (2.96)	708 (3.81)	
*20–34*	130 745 (88.27)	110 342 (88.43)	20 403 (87.37)	
*35–49*	14 771 (8.63)	12 151 (8.60)	2620 (8.82)	
Marital status				<0.05
*Never-married/widowed/divorced/separated*	2197 (1.16)	1755 (1.17)	442 (1.08)	
*Married*	147 425 (98.84)	124 136 (98.83)	23 289 (98.92)	
Current age of child in months				≥0.05
*<6*	18 516 (12.19)	15 635 (12.19)	2881 (12.20)	
*6–23*	54 769 (36.89)	46 167 (36.84)	8602 (37.18)	
*≥24*	76 337 (50.92)	64 089 (50.97)	12 248 (50.62)	
Sex of child				<0.05
*Male*	80 366 (53.87)	67 836 (54.04)	12 530 (52.91)	
*Female*	69 256 (46.13)	58 055 (45.96)	11 201 (47.09)	
**Socioeconomic characteristics**				
Education				<0.05
*No education*	25 850 (16.43)	20 580 (15.20)	5270 (23.37)	
*Primary*	16 996 (11.14)	14 073 (10.90)	2923 (12.49)	
*Secondary*	81 615 (53.43)	69 203 (54.08)	12 412 (49.78)	
*Higher*	25 161 (19.00)	22 035 (19.82)	3126 (14.35)	
Household wealth				<0.05
*Poorest*	31 912 (19.50)	25 319 (17.85)	6593 (28.78)	
*Poorer*	33 557 (20.58)	27 540 (19.90)	6017 (24.40)	
*Middle*	30 846 (20.33)	26 127 (20.64)	4719 (18.57)	
*Richer*	28 721 (20.52)	24 825 (21.26)	3896 (16.34)	
*Richest*	24 586 (19.07)	22 080 (20.35)	2506 (11.91)	
Health insurance				<0.05
*No*	107 711 (75.41)	89 746 (74.44)	17 965 (80.87)	
*Yes*	41 911 (24.59)	36 145 (25.56)	5766 (19.13)	
Type of residence				<0.05
*Urban*	34 635 (29.76)	29 987 (30.87)	4648 (23.52)	
*Rural*	114 987 (70.24)	95 904 (69.13)	19 083 (76.48)	
Religion				<0.05
*Hindu*	113 329 (80.46)	96 883 (80.69)	16 446 (79.15)	
*Muslim*	20 941 (15.11)	17 339 (14.78)	3602 (16.99)	
*Christian*	8996 (1.97)	6536 (1.99)	2460 (1.83)	
*Others*	6356 (2.46)	5133 (2.53)	1223 (2.04)	
**Pregnancy-related characteristics**				
Birth order number				<0.05
*1st*	54 060 (36.39)	45 983 (36.89)	8077 (55.71)	
*2nd*	54 633 (37.42)	46 433 (37.85)	8200 (32.28)	
*≥3rd*	40 929 (26.19)	33 475 (25.26)	7454 (12.01)	
Antenatal visits				<0.05
*<4*	57 210 (37.60)	44 379 (34.38)	12 831 (55.71)	
*4–7*	62 631 (41.16)	54 706 (42.74)	7925 (32.28)	
*≥8*	29 781 (21.24)	26 806 (22.88)	2975 (12.01)	
Place of delivery				<0.05
*Public facility*	110 797 (68.71)	91 639 (67.47)	19 158 (75.73)	
*Private facility*	38 825 (31.29)	34 252 (32.53)	4573 (24.27)	
**Total**	149 622 (100)	125 891 (84.92)	23 731 (15.08)	

PNC – postnatal care

*Two-sample χ^2^ tests

### Determinants of PNC utilisation

In the fully adjusted model, the odds of mothers receiving PNC were 21% greater for those with higher education (OR = 1.21; 95% CI = 1.13, 1.30), 27% greater for those from the richest quintile (OR = 1.27; 95% CI = 1.17, 1.37), and 19% greater for those with health insurance (OR = 1.19; 95% CI = 1.14, 1.24) compared to their respective reference group (**Table 3**). The association between pregnancy-related variables and mother’s PNC utilisation was also statistically significant. Mothers who had eight or more ANC visits (OR = 1.97; 95% CI = 1.86, 2.09) and those who delivered at private facilities (OR = 1.28; 95% CI = 1.22, 1.34) were more likely to receive PNC utilisation.

**Table 3:** Adjusted odds ratios of PNC utilisation by demographic, socioeconomic, and pregnancy-related variables from multilevel logistic regression in India, NFHS 2019–21

	Mother's PNC, OR (95% CI)*	Newborn's PNC, OR (95% CI)*
**Demographic characteristics**		
Maternal age in years		
*15–19*	1 (base)	1 (base)
*20–34*	1.11 (1.01, 1.22)†	1.00 (0.90, 1.11)
*35–49*	1.13 (1.01, 1.27)†	1.02 (0.90, 1.14)
Marital status		
*Never, married/widowed/divorced/separated*	1 (base)	1 (base)
*Married*	0.96 (0.84, 1.09)	1.05 (0.92, 1.20)
Current age of child in months		
*<6*	1 (base)	1 (base)
*6–23*	0.98 (0.93, 1.03)	1.01 (0.95, 1.07)
*≥24*	0.94 (0.89, 0.99)†	0.96 (0.91, 1.01)
Sex of child		
*Male*	1 (base)	1 (base)
*Female*	0.98 (0.95, 1.02)	0.96 (0.93, 1.00)†
**Socioeconomic characteristics**		
Education		
*No education*	1 (base)	1 (base)
*Primary*	1.11 (1.05, 1.18)†	1.16 (1.09, 1.24)†
*Secondary*	1.20 (1.14, 1.26)†	1.20 (1.14, 1.26)†
*Higher*	1.21 (1.13, 1.30)†	1.22 (1.13, 1.31)†
Household wealth		
*Poorest*	1 (base)	1 (base)
*Poorer*	1.05 (1.00, 1.10)†	1.07 (1.02, 1.13)†
*Middle*	1.13 (1.07, 1.19)†	1.13 (1.07, 1.20)†
*Richer*	1.14 (1.07, 1.22)†	1.12 (1.05, 1.20)†
*Richest*	1.27 (1.17, 1.37)†	1.30 (1.19, 1.41)†
Health insurance		
*No*	1 (base)	1 (base)
*Yes*	1.19 (1.14, 1.24)†	1.15 (1.10, 1.20)†
Type of residence		
*Urban*	1 (base)	1 (base)
*Rural*	1.03 (0.97, 1.09)	1.02 (0.96, 1.09)
Religion		
*Hindu*	1 (base)	1 (base)
*Muslim*	1.01 (0.95, 1.08)	0.98 (0.92, 1.04)
*Christian*	0.95 (0.84, 1.07)	0.95 (0.84, 1.07)
*Others*	0.89 (0.80, 1.00)†	0.94 (0.84, 1.05)
**Pregnancy, related characteristics**		
Birth order number		
*1st*	1 (base)	1 (base)
*2nd*	1.01 (0.97, 1.05)	0.98 (0.94, 1.02)
*≥3rd*	1.01 (0.97, 1.06)	1.02 (0.97, 1.07)
Antenatal visits		
*<4*	1 (base)	1 (base)
*4–7*	1.73 (1.66, 1.79)†	1.76 (1.69, 1.83)†
*≥8*	1.97 (1.86, 2.09)†	1.96 (1.84, 2.08)†
Place of delivery		
*Public facility*	1 (base)	1 (base)
*Private facility*	1.28 (1.22, 1.34)†	1.20 (1.15, 1.26)†

CI – confidence interval, OR – odds ratio, PNC – postnatal care

*Adjusted for maternal age, marital status, age of child, sex of child, maternal education, household wealth, health insurance, type of residence, religion, birth order number, ANC visit, and place of delivery.

†*P*-value <0.05 per two-sample tests.

Similarly, the probability of newborns receiving PNC was statistically significantly associated with selected socioeconomic characteristics. In the fully adjusted model, the odds of newborns receiving PNC were 22% greater for those with higher level of maternal education (OR = 1.22; 95% CI = 1.13, 1.31), 30% higher for those belonging to the richest quintile (OR = 1.30; 95% CI = 1.19, 1.41), and 15% higher for those whose mothers had health insurance (OR = 1.15; 95% CI = 1.10, 1.20) compared to their respective reference group. Some pregnancy-related variables were also significantly associated with newborn’s PNC utilisation: having eight or more ANC visits (OR = 1.96; 95% CI = 1.84, 2.08) and delivering in private health facilities (OR = 1.20; 95% CI = 1.15, 1.26) (**Table 3**).

### Partitioning geographic variation in PNC utilisation

In the null model, 56.64% of the total geographic variation for mothers’ PNC utilisation was attributable to clusters, 26.06% to states/union territories, and 17.30% to districts (**Table 4**). Similar partitioning patterns remained after adjusting for all the demographic, socioeconomic, and pregnancy-related characteristics. The geographic variation in newborns’ PNC utilisation was a little larger at the state level (31.15%), although most of the variation was still attributed to clusters (53.95%) in the null model (**Table 5**).

**Table 4:** Variance estimates in logit scale (95% CI) and variance explained in mother’s PNC utilisation at state, district, and cluster levels from multilevel logistic regressions, NFHS 2019–21

	Model 1*	Model 2†	Model 3‡	Model 4§
**Level 4: State (n = 36)**				
Variance estimate (95% CI)	0.486 (0.238, 0.735)	0.484 (0.237, 0.732)	0.386 (0.185, 0.587)	0.320 (0.152, 0.488)
VPC (%)‖	26.06	25.95	21.87	19.14
Variance explained (%)¶		0.40	16.10	26.60
**Level 3: District (n = 707)**				
Variance estimate (95% CI)	0.323 (0.278, 0.368)	0.323 (0.279, 0.368)	0.313 (0.270, 0.357)	0.290 (0.249, 0.331)
VPC (%)‖	17.30	17.32	17.75	17.36
Variance explained (%)¶		−0.10	−2.60	−0.30
**Level 2: Cluster (n = 29 455)**				
Variance estimate (95% CI)	1.057 (1.008, 1.106)	1.058 (1.009, 1.107)	1.066 (1.017, 1.115)	1.061 (1.012, 1.110)
VPC (%)‖	56.64	56.72	60.38	63.50
Variance explained (%)¶		−0.10	−6.60	−12.10

CI – confidence interval, VPC – variance partitioning coefficient

*Model 1: Null model.

†Model 2: Adjusted for maternal age, marital status, age of child, sex of child.

‡Model 3: Model 2 and additional adjustments for maternal education, household wealth, health insurance, type of residence, religion.

§Model 4: Model 3 and additional adjustments for birth order number, ANC visit, place of delivery.

‖Proportion of total geographic variation attributable to each level. See Equation S1 in the **Online Supplementary Document**.

¶Change in variance estimates from model 1 to model 2 (or model 3 or model 4). See Equation S2 in the **Online Supplementary Document**.

**Table 5:** Variance estimates in logit scale (95% CI) and variance explained in newborn’s PNC utilisation at state, district, and cluster levels from multilevel logistic regressions, NFHS 2019–21

	Model 1*	Model 2†	Model 3‡	Model 4§
**Level 4: State (n = 36)**				
Variance estimate (95% CI)	0.665 (0.332, 0.998)	0.664 (0.330, 0.998)	0.580 (0.286, 0.873)	0.512 (0.252, 0.773)
VPC (%)‖	31.15	31.02	28.14	26.09
Variance explained (%)¶		0.40	9.70	16.20
**Level 3: District (n = 707)**				
Variance estimate (95% CI)	0.318 (0.273, 0.363)	0.320 (0.274,0.366)	0.310 (0.265,0.354)	0.288 (0.246,0.330)
VPC (%)‖	14.91	14.93	15.03	14.67
Variance explained (%)¶		−0.10	−0.80	1.60
**Level 2: Cluster (n = 29 455)**				
Variance estimate (95% CI)	1.151 (1.098, 1.205)	1.157 (1.103, 1.211)	1.171 (1.116, 1.225)	1.164 (1.109, 1.218)
VPC (%)‖	53.95	54.04	56.83	59.24
Variance explained (%)¶		−0.20	−5.30	−9.80

CI – confidence interval, VPC – variance partitioning coefficient

*Model 1: Null model.

†Model 2: Adjusted for maternal age, marital status, age of child, and sex of child.

‡Model 3: Model 2 and additional adjustments maternal education, household wealth, health insurance, type of residence, religion.

§Model 4: Model 3 and additional adjustments birth order number, ANC visit, place of delivery.

‖Proportion of total geographic variation attributable to each level. See Equation S1 in the **Online Supplementary Document**.

¶Change in variance estimates from model 1 to model 2 (or model 3 or model 4). See Equation S2 in the **Online Supplementary Document**.

### Explaining variation in PNC utilisation

In comparing variance estimates from the cumulative inclusion of covariates, we found that demographic factors failed to explain variations in mother’s PNC utilisation at all levels (<1%) (**Table 4**). Further adjustment for socioeconomic factors explained 16% of the between-state variance of mother’s PNC utilisation, but none of the between-district and between-cluster variance. Meanwhile, 27% of between-state variation was explained in the fully adjusted model (i.e. state level variance estimate reduced from 0.486 (95% CI = 0.238, 0.735) to 0.320 (95% CI = 0.152, 0.488)). However, the same set of covariates did not explain any of the between-district variation in mother’s PNC utilisation. In fact, the between-cluster variation even increased by 12% after covariate adjustment. We found similar results for newborn’s PNC utilisation. Approximately 9.70% of between-state variance in newborn’s PNC utilisation was explained by demographic and socioeconomic factors, whereas none was explained for between-district and between-cluster variance (**Table 5**). Variance estimate at the state level decreased from 0.665 (95% CI = 0.332, 0.998) to 0.512 (95% CI = 0.252, 0.773) after adjusting for all demographic, socioeconomic, and pregnancy-related factors. However, variance estimates at the cluster level increased from 1.151 (95% CI = 1.098, 1.205) to 1.164 (95% CI = 1.109, 1.218) in the full model, compared to the null model.

### State-specific analyses

**Figure 1:**
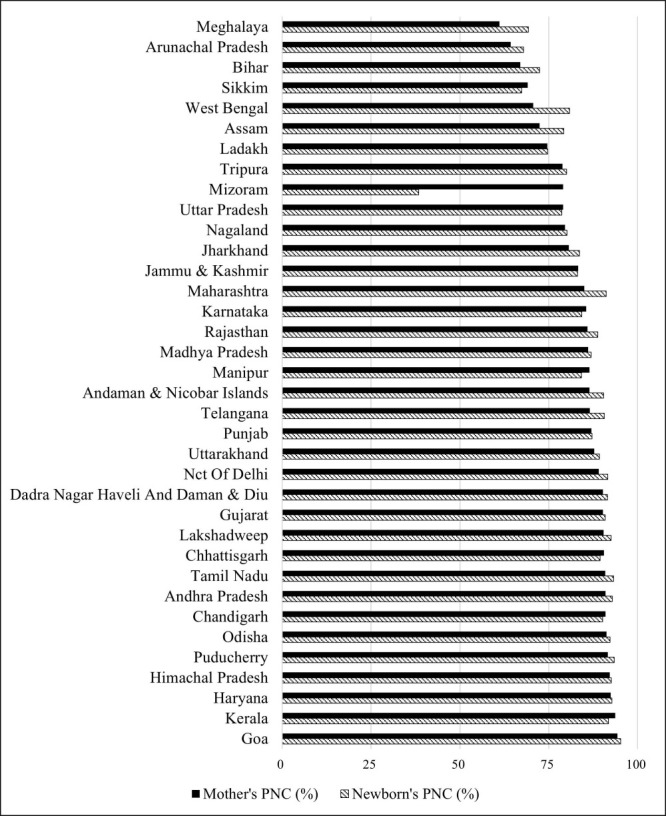
Percent of PNC utilisation across 36 states/union territories of India, NFHS 2019–21. NHFS – National Family Health Survey, PNC – postnatal care.

The prevalence of PNC utilisation in India varied widely across the 36 states/union territories, ranging from 61.09% (Meghalaya) to 94.31% (Goa) for mothers, and from 38.4% (Mizoram) to 95.20% (Goa) for newborns (**Figure 1**, Table S1 in **Online Supplementary Document**). The VPC for districts and clusters from state-specific analyses of three-level models showed that the clusters consistently account for more than half of the total geographic variations in PNC utilisation for both mothers (ranging from 58.80% in Meghalaya to 98.59% in Nagaland) and newborns (ranging from 67.53% in Bihar to 98.89% in Punjab) in the null model (**Table 6**, **Table 7**). Despite the majority of geographic variance in the PNC utilisation being attributed to the clusters, the percent explained by demographic, socioeconomic, and pregnancy-related factors remained low across all states. The same set of covariates explained up to almost 30% of the between-district variance in mother's PNC utilisation across all states, except for Andhra Pradesh, Chhattisgarh, Haryana, Nagaland, Odisha, Telangana, Uttarakhand, and West Bengal (**Table 6**). For the newborn's PNC utilisation, up to 99.90% and 39.30% of the between-district variation in Punjab and Assam, respectively, were explained by covariates (**Table 7**).

**Table 6:** Proportion of total variation in mother’s PNC utilisation within 24 h attributable to cluster and district levels from state-specific models in India, NFHS 2019–21

	Null model, VPC (%)*		Full model, variance explained (%)†‡	
**States§**	**District**	**Cluster**	**District**	**Cluster**
Andhra Pradesh	7.32	92.68	−22.30	1.80
Arunachal Pradesh	35.91	64.09	7.90	−4.40
Assam	8.80	91.20	28.70	−2.80
Bihar	37.49	62.51	6.20	−3.70
Chhattisgarh	13.56	86.44	−11.60	1.80
Gujarat	28.08	71.92	12.80	−5.00
Haryana	16.99	83.01	−7.70	1.60
Jammu & Kashmir	21.18	78.82	18.00	−4.80
Jharkhand	10.15	89.85	22.70	−2.60
Karnataka	21.04	78.96	12.60	−3.40
Madhya Pradesh	22.19	77.81	9.00	−2.60
Maharashtra	26.36	73.64	16.80	−6.00
Manipur	7.16	92.84	24.80	−1.90
Meghalaya	41.20	58.80	0.40	−0.30
Nagaland	1.41	98.59	−1.40	0.00
Nct Of Delhi	15.99	84.01	25.30	−4.80
Odisha	6.33	93.67	−7.90	0.50
Punjab	7.97	92.03	15.20	−1.30
Rajasthan	17.65	82.35	3.60	−0.80
Tamil Nadu	7.53	92.47	6.40	−0.50
Telangana	13.81	86.19	−0.40	0.10
Tripura	24.55	75.45	17.50	−5.70
Uttar Pradesh	29.25	70.75	8.50	−3.50
Uttarakhand	15.25	84.75	−5.70	1.00
West Bengal	35.21	64.79	−2.80	1.50

VPC – variance partitioning coefficient

*Proportion of total geographic variation attributable to each level. See Equation S1 in the **Online Supplementary Document**.

†Full model: Adjusted for maternal age, marital status, age of child, sex of child, maternal education, household wealth, health insurance, type of residence, religion, birth order number, ANC visit, and place of delivery.

‡Change in variance estimates from null model to full model. See Equation S2 in the **Online Supplementary Document**.

§We excluded Andaman and Nicobar Islands, Chandigarh, Goa, Himachal Pradesh, Kerala, Ladakh, Lakshadweep, Mizoram, Dadra and Nagar Haveli and Daman and Diu, Puducherry, and Sikkim.

**Table 7:** Proportion of total variation in newborn’s PNC utilisation within 24 h attributable to cluster and district levels from state-specific models in India, NFHS 2019–21

	Null model, VPC (%)*		Full model, variance explained (%)†‡	
**States§**	**District**	**Cluster**	**District§**	**Cluster**
Arunachal Pradesh	31.53	68.47	9.60	−4.40
Assam	8.67	91.33	39.30	−3.70
Bihar	32.47	67.53	5.90	−2.80
Chhattisgarh	19.59	80.41	−7.10	1.70
Gujarat	25.07	74.93	11.70	−3.90
Haryana	20.93	79.07	3.10	−0.80
Jammu & Kashmir	18.02	81.98	11.70	−2.60
Jharkhand	11.66	88.34	24.10	−3.20
Karnataka	10.69	89.31	15.20	−1.80
Madhya Pradesh	16.20	83.80	9.30	−1.80
Maharashtra	26.31	73.69	10.40	−3.70
Manipur	16.34	83.66	2.40	−0.50
Meghalaya	26.28	73.72	−1.20	0.40
Mizoram	8.39	91.61	−54.10	5.00
Nagaland	10.11	89.89	−21.50	2.40
Nct Of Delhi	9.29	90.71	−12.90	1.30
Odisha	5.62	94.38	−0.10	0.00
Punjab	1.11	98.89	99.90	−1.10
Rajasthan	18.29	81.71	5.30	−1.20
Tamil Nadu	3.78	96.22	−26.00	1.00
Telangana	5.42	94.58	−0.30	0.00
Tripura	28.49	71.51	−1.60	0.70
Uttar Pradesh	31.43	68.57	8.50	−3.90
Uttarakhand	7.00	93.00	−44.00	3.30
West Bengal	29.19	70.81	4.40	−1.80

VPC – variance partitioning coefficient

*Proportion of total geographic variation attributable to each level. See Equation S1 in the **Online Supplementary Document**.

†Full model: Adjusted for maternal age, marital status, age of child, sex of child, maternal education, household wealth, health insurance, type of residence, religion, birth order number, ANC visit, and place of delivery.

‡Change in variance estimates from null model to full model. See Equation S2 in the **Online Supplementary Document**.

§We excluded Andaman and Nicobar Islands, Chandigarh, Goa, Himachal Pradesh, Kerala, Ladakh, Lakshadweep, Mizoram, Dadra and Nagar Haveli and Daman and Diu, Puducherry, and Sikkim.

## DISCUSSION

Our study based on the latest nationally representative data from India has several important findings. From the variance partitioning analyses, we found that most geographic variation in PNC utilisation was attributable to clusters, followed by states and districts. We also found that cluster variation was substantial across all selected states. A recent study that attempted to partition the geographic variation in a composite score of maternal health care quality in India had similar results (VPC for clusters = 58.3%, states/union territories = 29.3%, and districts = 12.4%), indicating the relative importance of clusters in reducing geographic inequalities for overall maternal health care quality in India [18]. Although the Indian government adopted the Reproductive, Maternal, Newborn, Child, and Adolescent Health (RMNCH^+^A) framework to improve maternal and child health since 2013 [46], the intervention undertaken within have mainly been focussed at the district level [29,47,48]. For example, the Labour room Quality improvement Initiative (LaQshya) launched in 2017 aimed to improve and ensure the quality of care during antenatal and immediate postnatal periods in all government medical college hospitals and high case-load district hospitals by offering practical training on clinical protocols across districts [48]. The extensive variation in PNC utilisation found within a district and across clusters clearly suggests the necessity of the RMNCH^+^A framework to be organised and adapted at the local level. For example, the LaQshya could evaluate the quality of care and provide training programmes at cluster-level health facilities, such as sub-centre. Similarly, other ongoing programmes to improve PNC utilisation should consider the substantial within-district, between-cluster variation.

To further advance the accumulating literature quantifying small area variation in various health outcomes in India [15–18,49], we assessed the extent to which total contextual variation in PNC utilisation at each geographic level can be explained by different compositional factors. When we simultaneously considered various demographic, socioeconomic, and pregnancy-related characteristics, we found maternal education, household wealth, health insurance, ANC visits, and delivery at private facilities to be statistically significant factors associated with maternal and newborn PNC utilisation. This finding aligns with previous studies from low- and middle-income countries (LMICs), where a higher maternal education level was consistently reported to be associated with higher probabilities of ANC visits and delivery in health facilities due to improved cognitive abilities (e.g. health literacy), economic opportunities, and women’s autonomy in utilising appropriate health care services [36,50–52]. For similar reasons, it is reasonable to expect higher socioeconomic status to induce PNC utilisation [36], which is what we found in the context of India. Mothers with health insurance were also more likely to utilise PNC services, which can be explained by the reduction in financial burden, especially for poor households, thereby increasing their access to health care [39].

We also found significant relationships between ANC visits and PNC utilisation, even after adjusting for other characteristics. This can be explained by the continuum of care framework, outlining the subsequent transition from ANC visits to PNC utilisation throughout the maternity cycle [53]. Based on this framework, frequent ANC contacts with health personnel were found to enable adequate counselling and information provision related to post-delivery care in rural Tanzania [54]. Similarly, mothers who made four or more ANC visits had a ten times higher probability of PNC utilisation in Ethiopia [40]. In the context of India, we found that eight or more ANC visits (i.e. per the updated WHO guidelines) increased PNC utilisation even more than four or more visits, and that the same association was also true for newborns. Delivery at public vs private facilities also mattered, as previously found in LMICs [55,56]. For example, mothers who delivered at public facilities were almost twice more likely to not use PNC compared to mothers who delivered at private facilities in Palestine, which was attributed to better medical resources and individualised care provided to patients in private facilities [55]. Similarly, a prior study using nationally representative data of India in 2015–16 also reported that mothers who delivered at private facilities were likely to remain in the institutions longer than those who delivered at public facilities, enabling more frequent postnatal examinations and less birth complications [56].

In addition to the significant on average associations, we found that compositional effects of socioeconomic and pregnancy-related factors were substantial in explaining the variability at the state level, implying that statewide inequalities in PNC utilisation may be driven by the clustering of individuals with similar SES characteristics. However, we found the clustering effect of demographic factors to be minimal and relatively insignificant in explaining the statewide differences in PNC utilisation. While a previous attempt to partition statewide inequalities in ANC and PNC utilisation in India has found socioeconomic characteristics to be important [57], we applied a multilevel perspective to explain variability at multiple geographic levels simultaneously. To what extent this finding is generalisable to other countries should be explored in future multilevel studies considering the unique geographic, administrative, and policy relevant units in the given context.

Despite the large geographic variation in PNC utilisation across clusters, almost none were explained by compositional effects across all states. This may indicate the stronger role of contextual factors at the cluster level, such as the availability of high-quality health facilities. For example, distance to health facilities [58] and suboptimal quality of the services at the community level [59] are identified as barriers to delivery at health facilities in India. While the lack of data on such contextual measures prohibited us from examining their role here, future studies should investigate contextual mechanisms – and their interaction with individual characteristics – to better explain between-cluster variation in PNC utilisation.

There are a few limitations to this study. First, our study may be subject to reporting bias, as the responses to the outcomes and covariates were all self-reported by mothers. For pregnancy-related variables, including PNC utilisation, the mothers were asked to report information on the births that occurred in the past five years preceding the survey. Therefore, they may not have correctly remembered the exact timing of PNC check-ups. However, recent research conducted in Bangladesh, Cambodia, and Kenya found mothers’ recall on coverage and quality of maternal interventions (e.g. ANC and PNC) to be valid with high accuracy, regardless of maternal education level [60]. Second, there might be selection bias in our findings due to the inclusion criteria. For example, we excluded newborns who were not alive at the time of the survey and those who did not answer on demographic and socioeconomic characteristics that could have influenced their PNC utilisation. However, the comparison between the final study sample and the original sample presented only minimal differences in terms of demographic and socioeconomic characteristics, suggesting a low possibility of selection bias (data not shown).

Third, although the administrative role and geographic size of villages in rural areas and blocks in urban areas are not identical, we treated them as the same unit of ‘clusters’ to reflect the local geographic level lower than districts. Fourth, variance partitioning in any multilevel analysis is highly sensitive to the inclusion and exclusion of relevant random effects [61]. For example, other geographic levels we did not consider here due to lack of geographic identifiers (e.g. divisions, subdistricts) may have a practical impact on PNC utilisation. Nevertheless, we made the best use of the existing database based on evidence from prior multilevel studies conducted in the context of India [17–21,43]. Lastly, the absence of data for specific elements of PNC examination prohibited further investigation into utilisation patterns of essential examinations needed for mothers and newborns. PNC-related data in the latest DHS (7th round) are restricted to questions on whether mothers and newborns had postnatal utilisations, when and where their first utilisation occurred, who provided PNC, and whether newborns were placed skin-to-skin with their mothers, weighed at delivery, and received vaccinations [62]. This enables evaluation of only three out of 11 components and 10 components necessary for mothers’ and newborns’ PNC examination, respectively, based on the WHO guidelines [3]. For evidence-based policy discussion to improve PNC utilisation, surveys conducted in LMICs should expand their questionnaire to collect more detailed information about PNC coverage.

## CONCLUSIONS

Using the latest nationally representative data, we conducted the first ever study on the compositional vs contextual effects on PNC utilisation among mothers and newborns in India. Our finding on the relative importance of cluster variation in PNC utilisation, which was only partially explained by compositional effects of demographic, socioeconomic, and pregnancy-related factors, has important implications for future studies and policies that aim to improve maternal and child health in India. From the policy perspective, local variability in PNC utilisation within districts should be considered in identifying the population with the greatest need for the services. Moreover, current maternal health interventions focusing solely on individual characteristics, such as mother’s financial resources (e.g. the Janani Suraksha Yojana programme), may be effective in reducing statewide inequalities of PNC utilisation, but not necessarily for local variability. Improvement in the geographic disparities of PNC utilisation can contribute to the progress on SDG targets in reducing maternal and neonatal mortality, both in India and globally.

## Additional material


Online Supplementary Document
