# Supplementary material for: Multilevel analysis of determinants in postnatal care utilisation among mother-newborn pairs in India, 2019–21
Source: J Glob Health. 2024 May 10;14:04085. doi: 10.7189/jogh.14.04085 (PMC11079700; doi:10.7189/jogh.14.04085)
Supplement: Online Supplementary Document [file jogh-14-04085-s001.pdf]

Online Supplementary Documents

Figure S1. Construction of the analytic sample of mother-newborn pairs, NFHS 2019-21

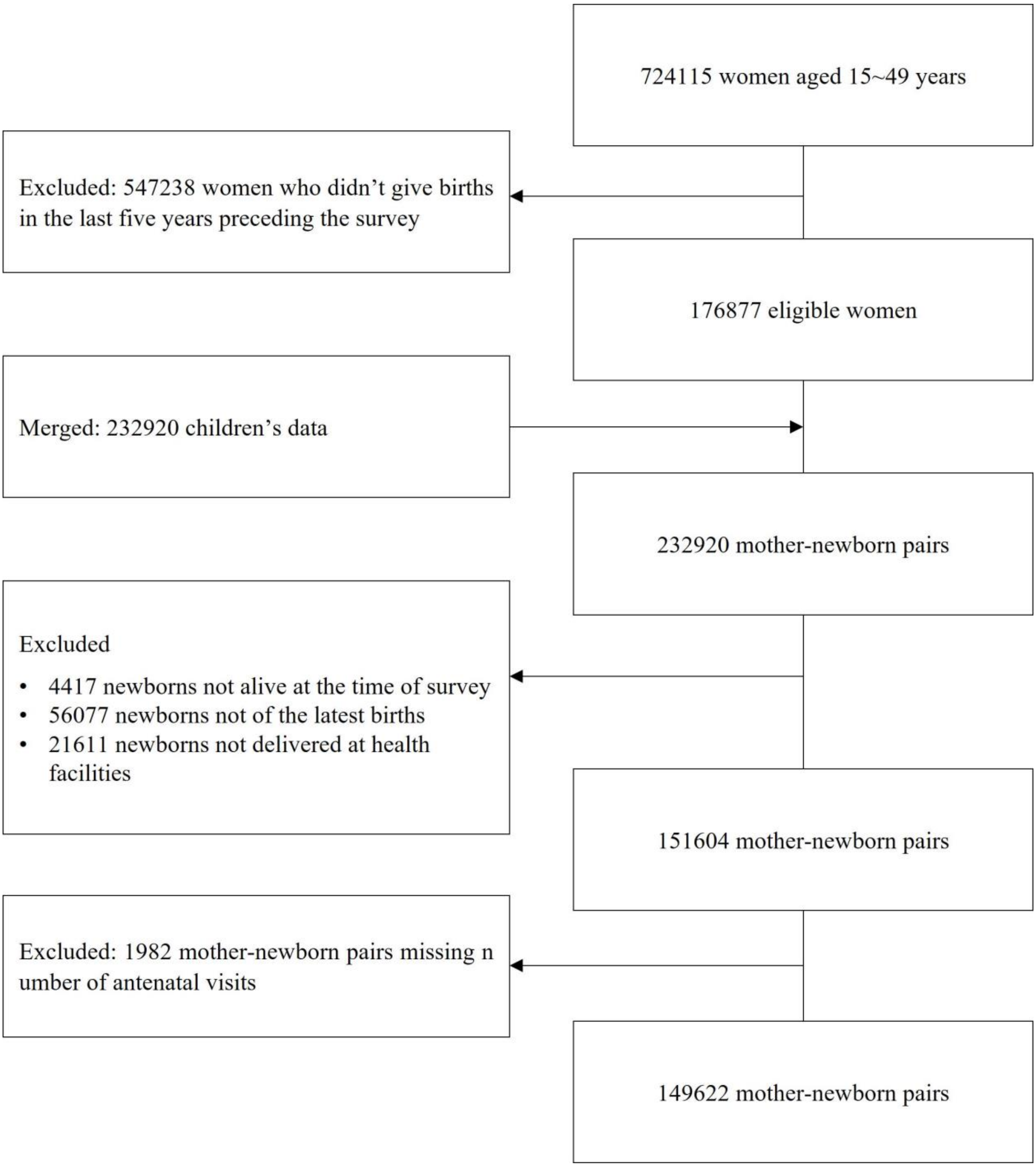

Table S1. Distribution of number of districts, clusters, and individuals, and proportion of PNC utilization, within 36 Indian states/union territories, NFHS 2019-21

States	# Districts	# Clusters	# Individuals	% Mother's PNC	% Newborn's PNC
Andaman and Nicobar Islands	3	117	380	86.45	90.42
Andhra Pradesh	13	531	1986	90.97	92.88
Arunachal Pradesh	20	815	3505	64.30	67.88
Assam	33	1373	7671	72.41	79.24
Bihar	38	1699	10475	67.03	72.46
Chandigarh	1	36	135	90.99	90.11
Chhattisgarh	27	1113	5325	90.55	89.41
Goa	2	80	317	94.31	95.20
Gujarat	33	1370	6889	90.23	90.89
Haryana	22	869	4777	92.44	92.82
Himachal Pradesh	12	484	1833	92.18	92.59
Jammu and Kashmir	20	832	4383	83.31	83.09
Jharkhand	24	1058	5558	80.67	83.66
Karnataka	30	1233	6110	85.54	84.29
Kerala	14	559	2146	93.71	91.74
Ladakh	2	80	422	74.60	74.66
Lakshadweep	1	42	243	90.40	92.50
Madhya Pradesh	51	2085	10273	86.09	86.90
Maharashtra	36	1459	6713	84.98	91.16
Manipur	9	355	1749	86.43	84.16
Meghalaya	11	441	2582	61.09	69.26
Mizoram	8	310	1375	79.04	38.44
Nagaland	11	366	909	79.61	80.11
Dadra and Nagar Haveli and Daman and Diu	3	123	601	90.23	91.52
Nct Of Delhi	11	443	2123	89.07	91.62
Odisha	30	1253	6405	91.23	92.27
Puducherry	4	162	608	91.58	93.45
Punjab	22	900	4079	87.02	87.17
Rajasthan	33	1482	10128	85.90	88.83
Sikkim	4	155	528	69.05	67.32
Tamil Nadu	32	1303	5127	90.91	93.23
Telangana	31	1269	5139	86.58	90.63
Tripura	8	335	1554	78.85	80.06
Uttar Pradesh	75	3343	20871	79.06	78.66
Uttarakhand	13	557	2422	87.76	89.27
West Bengal	20	823	4281	70.63	80.88

PNC – postnatal care, NHFS – national family health survey.

Equation S1. Calculating variance partitioning coefficient (VPC)

$$VPC = \frac{\sigma_z^2}{\sigma_{u0}^2 + \sigma_{v0}^2 + \sigma_{f0}^2} \quad \times 100$$

The proportion of variation in the log odds of PNC utilisation attributed to each level *z* was calculated by dividing variance at level *z* by the total geographic variance (σ_{u0}^2: between-cluster variance, σ_{v0}^2: between-district variance, σ_{f0}^2: between-state variance).

Equation S2. Calculating variance explained

$$Variance\ explained\ (\%) = \left(\frac{[\sigma_z^2]_{model\ 1} - [\sigma_z^2]_{model\ 2}}{[\sigma_z^2]_{model\ 1}}\right) \ \times 100$$

This equation was used to calculate variation in the log odds of PNC utilization at level *z* explained by model 2 compared to model 1.
